# Data supporting the role of enzymes and polysaccharides during cassava postharvest physiological deterioration

**DOI:** 10.1016/j.dib.2015.12.043

**Published:** 2016-01-06

**Authors:** Virgílio Gavicho Uarrota, Rodolfo Moresco, Eder Carlos Schmidt, Zenilda Laurita Bouzon, Eduardo da Costa Nunes, Enilto de Oliveira Neubert, Luiz Augusto Martins Peruch, Miguel Rocha, Marcelo Maraschin

**Affiliations:** a Federal University of Santa Catarina, Plant Science Center, Plant Morphogenesis and Biochemistry Laboratory, 1346 Admar Gonzaga Road, Florianópolis, SC 88034-001, Brazil; b Federal University of Santa Catarina, Laboratory of Plant Cell Biology, Centre for Biological Sciences, Department of Cell Biology, Embryology and Genetics, Florianópolis, SC 88040-900, Brazil; c Santa Catarina State Agricultural Research and Rural Extension Agency (EPAGRI), Experimental Station of Urussanga (EEUR), SC 446 Road, Km 19, Urussanga, SC 88840-000, Brazil; d Centre of Biological Engineering (CEB), University of Minho, Campus de Gualtar, 4710-057 Braga, Portugal

**Keywords:** PPD, Postharvest physiological deterioration, ROS, Reactive oxygen species, Supporting data, R software, Cassava, Enzymes, Deterioration

## Abstract

This data article is referred to the research article entitled *The role of ascorbate peroxidase, guaiacol peroxidase, and polysaccharides in cassava (Manihot esculenta Crantz) roots under postharvest physiological deterioration* by Uarrota et al. (2015). Food Chemistry 197, Part A, 737–746.

The stress duo to PPD of cassava roots leads to the formation of ROS which are extremely harmful and accelerates cassava spoiling. To prevent or alleviate injuries from ROS, plants have evolved antioxidant systems that include non-enzymatic and enzymatic defence systems such as ascorbate peroxidase, guaiacol peroxidase and polysaccharides. In this data article can be found a dataset called “newdata”, in RData format, with 60 observations and 06 variables. The first 02 variables (Samples and Cultivars) and the last 04, spectrophotometric data of ascorbate peroxidase, guaiacol peroxidase, tocopherol, total proteins and arcsined data of cassava PPD scoring. For further interpretation and analysis in R software, a report is also provided. Means of all variables and standard deviations are also provided in the Supplementary tables (“data.long3.RData, data.long4.RData and meansEnzymes.RData”), raw data of PPD scoring without transformation (PPDmeans.RData) and days of storage (days.RData) are also provided for data analysis reproducibility in R software.

**Specifications Table**

**Table:** 

Subject area	Chemistry, Biology
More specific subject area	Postharvest Biology
Type of data	Table in RData format
How data was acquired	Spectroscopy and laboratory PPD induction of cassava roots
Data format	Raw data
Experimental factors	Four cassava cultivars and 05 different times of root storage
Experimental features	PPD scoring, ascorbate peroxidase activity, guaiacol peroxidase activity, total proteins and tocopherol
Data source location	Florianópolis, Santa Catarina, Brazil, 27°35’48” S, 48°32’57” W
Data accessibility	Data available within this article

**Value of the data**

• Correlation of ascorbate peroxidase, guaiacol peroxidase, proteins and polysachharides with postharvest deterioration of cassava roots;
• Understand the role of enzymatic activities and polysaccharides in delaying cassava PPD;
• Use datasets and tutorials as benchmark for future multivariate analysis in R software.

## 1 Data

**Fig. 1:**
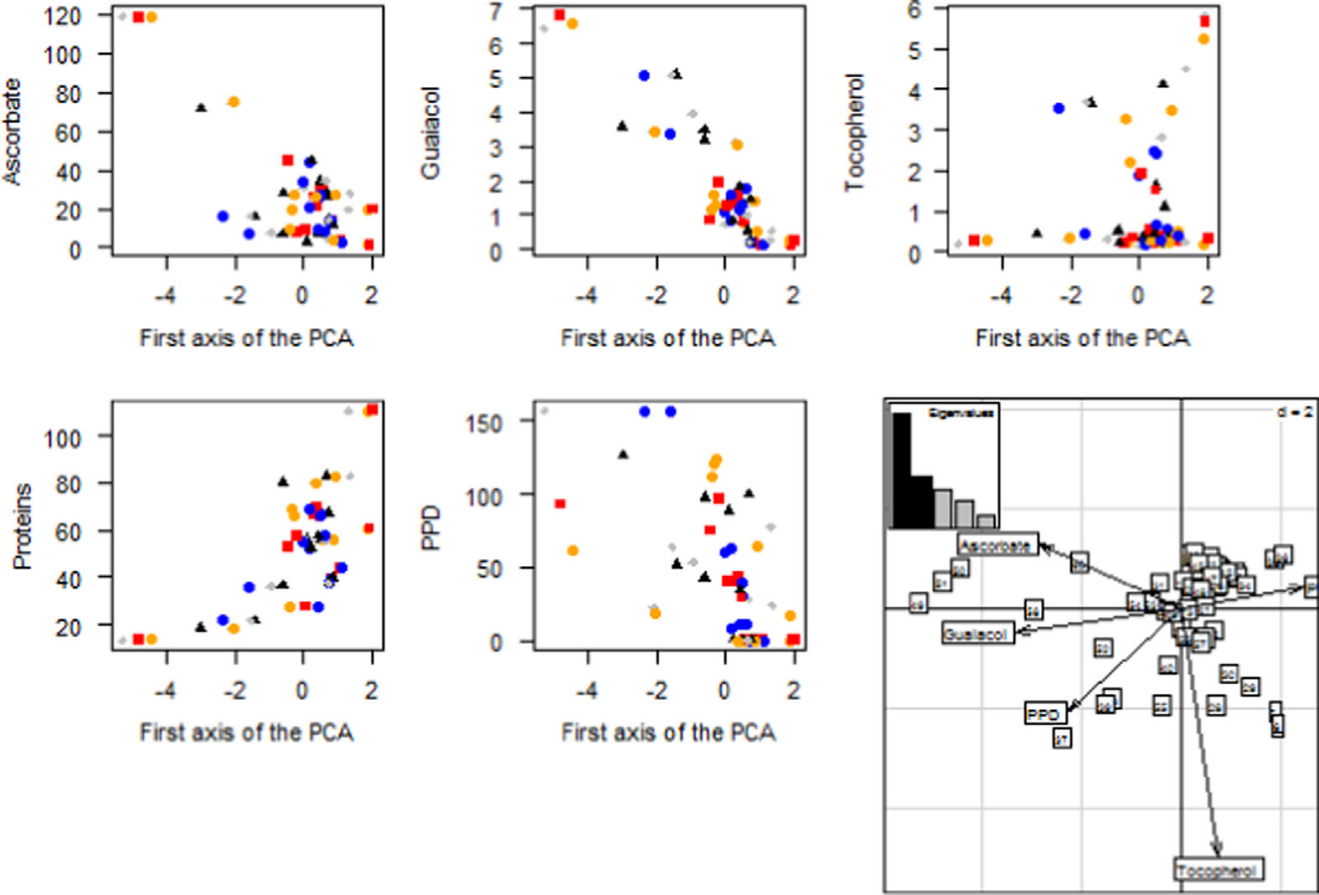
Variable importance in the first principal component and PCA analysis (right- last figure below) with the Eigen values and PCA loadings. Red colors represent fresh samples (non-stored), blue (samples stored during 3 days), black (5 days), gray (8 days) and orange (11 days of storage).

Dataset provided in this article represent the results of PPD induction of cassava roots, cultivated in Santa Catarina state, Brazil and spectrophotometric data of enzymatic activities during cassava storage (fresh samples and those stored during 3, 5, 8 and 11 days). Differently from those analysis described in the published manuscript, other analysis were performed such as linear discriminant analysis (LDA) and partial least square discriminant analysis (PLS-DA). Table 1 presents the results of model comparison. LDA model was the best with minor error rate of predicted samples. Fig. 1 shows the variable importance in the first principal analysis (A–D) and principal component analysis (PCA) together with the Eigen values and loadings.

## 2 Experimental design, materials and methods

Cassava cultivars were grown in Southern Brazil over the 2011/2012 growing season. Four cultivars were selected for this study, as follows: SCS 253 Sangão (hereinafter SAN), Branco (hereinafter BRA, a landrace), IAC576-70 (hereinafter IAC, a commercial variety), and Oriental (hereinafter ORI, a landrace). On-farm trials were carried out at the Ressacada Experimental Farm (Plant Science Center, Federal University of Santa Catarina, Florianópolis, SC, Brazil-27°35′48″ S, 48°32′57″ W) in September 2011, using the four cassava cultivars noted above. Samples of cassava cuttings for cultivation were provided by the Santa Catarina State Agricultural Research and Rural Extension Agency (EPAGRI) at Urussanga, the official state agriculture agency. The experimental design was in randomized blocks, with 4 blocks (6.3×15 m/block) spaced at 1 m. Each block consisted of four plots (12×1.2 m/plot) spaced at 0.5 m. Cassava cuttings (15 cm long) were planted upright and spaced at 1×1 m. Each plot was considered a treatment, and all crop management was mechanized. Chemical analysis of soil fertility was previously done, and cultivation was performed manually, following agroecological field handlings.

Cassava root samples (12 months old) were collected for analysis of non-stored samples and for induction of physiological deterioration under controlled conditions in the laboratory. Immediately after harvest, the roots were washed, proximal and distal parts of the root were removed, and cross sections were made (0.5–1 cm) over the remaining root, followed by storage at room temperature (66–76% humidity, 25 °C). Induction of PPD was performed for 11 days. Monitoring the progress of PPD and associated metabolic disturbances was performed daily after induction of PPD. Non-stored samples and those at 3, 5, 8, and 11 days after PPD induction were collected at each time point, dried (35–40 °C) in an oven, milled with a coffee grinder (Model DGC-20N series), and kept for analysis. For enzymatic analysis, fresh samples (batch of seven roots from each cultivar) were collected, grated using a food processor (Walita-Master Plus, Brazil), and stored (−80 °C) until analysis. Five independent experiments of PPD were carried out in which a randomized sampling of 3 sliced roots from each plant variety was scored (from 1–10% of PPD to 10–100% of PPD) over the 11-day experimental period. The information was imaged through a digital camera (OLYMPUS FE-4020, 14 megapixel), and the results were analyzed by visual inspection of the images. All enzymatic analysis were done as described in our manuscript (Uarrota et al., 2015).

## Figures and Tables

**Table 1:** Confusion matrix for LDA and PLS-DA models tested in our analysis.

	**Non-stored**		**3 days**		**5 days**		**8 days**		**11 days**	
**Samples**	**LDA**	**PLS-DA**	**LDA**	**PLS-DA**	**LDA**	**PLS-DA**	**LDA**	**PLS-DA**	**LDA**	**PLS-DA**
**Non-stored**	12.0	12.0	0.0	0.0	0.0	0.0	0.0	0.0	0.0	0.0
**3 days**	0.0	5.0	11.0	0.0	0.0	5.0	1.0	2.0	0.0	0.0
**5 days**	0.0	2.0	5.0	0.0	7.0	10.0	0.0	0.0	0.0	0.0
**8 days**	0.0	0.0	3.0	0.0	1.0	3.0	5.0	9.0	3.0	0.0
**11 days**	3.0	3.0	0.0	0.0	0.0	0.0	3.0	3.0	6.0	6.0

Error rate for LDA=0.3167.

Error rate for PLS-DA=0.383.
