# Supplementary material for: Data supporting the role of enzymes and polysaccharides during cassava postharvest physiological deterioration
Source: Data Brief. 2016 Jan 6;6:503–6. doi: 10.1016/j.dib.2015.12.043 (PMC4716459; doi:10.1016/j.dib.2015.12.043)
Supplement: Supplementary file 1 — Supplementary material [file mmc1.doc]

Conflict of interest

No one declared.
